# The 1 million words pathology report or the challenge of a reproducible and meaningful message

**DOI:** 10.1016/j.esmorw.2024.100044

**Published:** 2024-06-03

**Authors:** C. Eloy, P. Seegers, E. Bazyleva, F. Fraggetta

**Affiliations:** 1 Pathology Laboratory, Institute of Molecular Pathology and Immunology of University of Porto (IPATIMUP), Porto; 2 Pathology Department, Medical Faculty of University of Porto, Porto, Portugal; 3 Palga, National Pathology Databank, Houten, the Netherlands; 4 Belgian Society of Pathology, Brussels, Belgium; 5 Pathology Department, Gravina Hospital, Caltagirone, ASP Catania, Italy

**Keywords:** digital pathology, digital transformation, pathology report, computational pathology, synoptic reporting

## Abstract

Many years have passed since the pathology report was all about a single-sentence diagnosis based on morphology. The pathology report is an invaluable source of data that needs to evolve from a narrative reporting to a synoptic reporting system by standardizing data elements to ensure consistency and structured formats that improve completeness, interoperability, and scalability across different health care systems. The convergence of technology, structured data, and artificial intelligence propels the field of pathology toward a future where the synthesis of information benefits not only health care professionals and patients but also serves as a wellspring of knowledge for machines, paving the way for unprecedented strides in data mining and health care innovation.

## Highlights

• Pathology reports condense increasingly multiple source information.
• The amount of information may turn a pathology report into a confusing document.
• Technology is supporting the organization of data into synoptic reports.
• Artificial intelligence contributes to organizing data for the benefit of patient care.

Many years have passed since the pathology report was all about a single-sentence diagnosis based on morphology. The growing body of knowledge on each disease turned the pattern recognition activity without an integrated clinical and immunohistochemical/molecular context into an infertile diagnosis exercise, far from the precision medicine aimed at nowadays. The complex armamentarium for disease characterization that is now available to the modern pathologist also provides powerful inputs on evaluating prognosis and predictive markers that computational tools may even augment. The results emanating from all these sources must be organized, integrated, and rapidly managed in a readable, reproducible, and meaningful report. The challenge of reporting under these conditions requires training, knowledge of pitfalls and analytic issues, as well as a relative resistance to stressing elements.

The pathology report is an invaluable source of data that needs to evolve from a narrative reporting to a synoptic reporting system by standardizing data elements to ensure consistency and structured formats that improve completeness, interoperability, and scalability across different health care systems.^1^ The adequacy of the synoptic reporting is easily observed during multidisciplinary oncology meetings, transforming the analysis of the pathology report into an efficient and more accurate exercise that contributes to better-informed treatment decisions and consequent better patient outcomes.^2^

**Figure 1:**
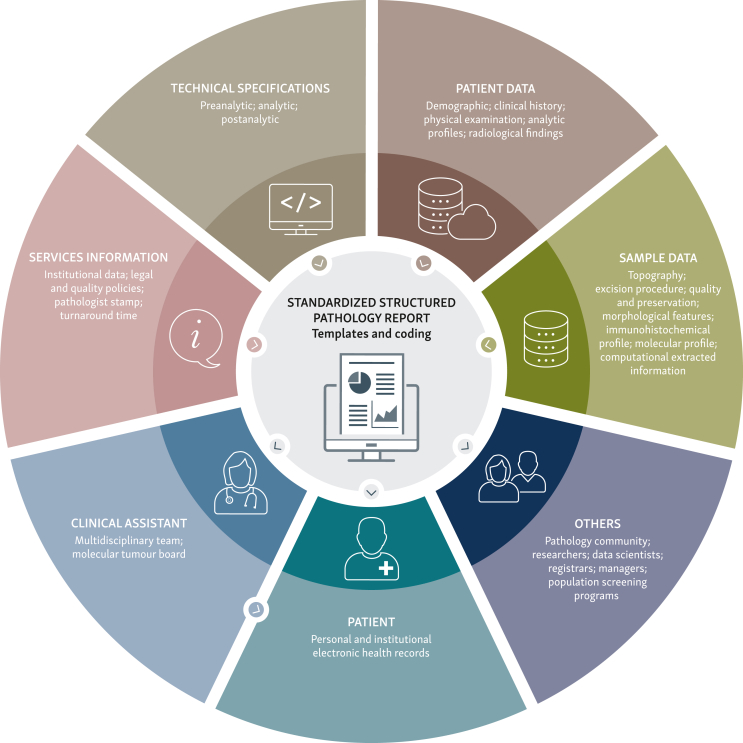
Information management at the pathology report level.

Today, pathologists are facing the challenge of translating a report with 1 million words into a reproducible and meaningful message, refining reporting methods and improving data-sharing standards, for the benefit of pathology pairs, clinicians, registrars, researchers, data scientists, quality control managers, and ultimately, patients.^3^^,^^4^ Although there are some attempts to standardize reporting, particularly in relation to cancer diagnosis, there is still room for imagination in the reporting of cytology and in the reporting of benign conditions. Coping with information overload can go over the limits in molecular pathology reporting due to the increasing volume of genomic data. Beyond standards in the interpretation and reporting of sequence variants in next-generation sequencing and whole genome sequencing, metadata, such as the quality and type of the specimen, combined with technical aspects, also contribute to the cumulative data to be reported.^5^^,^^6^ Questions on the reporting of molecular data with unproven clinical relevance or on conflicting pathologist–computer evaluation are also moot points still under discussion, that for now are generating extra data to include in the report as well. Studies suggest that formatting options such as column formats and shorter sentences contribute to better understanding and faster information retrieval, preventing overwhelming the users.^7^ In general, there is a need to compartmentalize information into subjects, separating the report about the sample from technical specifications, explanations, and disclaimers. The need to include in the report institutional data (name of the laboratory, management and responsibilities, quality control policy), technical specifications relevant to the exploitation of results/medico-legal purposes (description of the study, technical procedures, results of validation and performance tests, and others), and patient data in sufficient detail is also of paramount importance to avoid mislabelling while preserving confidentiality (Figure 1).

Recently, technology and the digital transformation of pathology laboratories^8^ have contributed to instigating the organization of data and respective reporting. Practical problems, related to the availability of specialized laboratory information systems (LISs) designed to facilitate standardization with a dedicated lexicon and speech recognition, are starting to be solved. Further, the convergence of informatics standards for producing reports in formats such as JavaScript Object Notation (JSON) or eXtensible Markup Language (XML) is contributing to interoperability and intraoperability.^7^ The integration of artificial intelligence (AI) tools, including natural language processing and large language models, into LISs is becoming increasingly common.^9^^,^^10^ These AI technologies can efficiently organize and translate information from unstructured pathology reports into structured formats. These structured data are then easily retrievable for statistical analysis. However, a significant challenge with these models lies in their dependency on the input content: information that is not included in the reports cannot be structured.^11^ Therefore the use of comprehensive synoptic reports is crucial. Such reports ensure that all necessary data are captured, preventing issues such as incomplete information, outdated classifications, or the omission of data critical for patient management. Ultimately, the goal is to go along with the patient movement, from institution to institution, from country to country, producing a reporting frame that is transversal at the national level or even at the international level. Generating synoptic reports that can be used at a national level is a complex process that encompasses activities on the range of regulations and laws, organizational policies, care processes, information requirements, applications, and informatics infrastructure.^12^

For each national activity regarding synoptic reporting, a jurisdiction frame under which the organization operates must be defined according to the regulations provided by governmental and supervisory authorities, including those at a European level such as the General Data Protection Regulation (GDPR) and European Health Data Space (EDHS). This frame will allow the organizational nation, as a general umbrella, or smaller organizational units, to establish their management policies to ensure all procedures for supporting the implementation of synoptic reporting. The maintenance of implemented synoptic reports, including core and noncore datasets, requires a centralized organization that depends on the chosen informatic application and on the information requirements that will incorporate the synoptic report itself. These information requirements consist of minimal datasets (core elements) inspired by international organizations such as the International Collaboration on Cancer Reporting (ICCR) or the College of American Pathologists (CAP), along with national guidelines. In addition, including the World Health Organization Classification of Tumours, the tumour–node–metastasis classifications, and international code systems, such as SNOMED Clinical Terms (SNOMED CT) or Logical Observation Identifiers Names and Codes (LOINC), for oncology is essential.^13^, ^14^, ^15^, ^16^, ^17^, ^18^, ^19^ The informatic application selected to manage this information will be storing, structuring, processing, analysing, or communicating information that are crucial factors for successfully adapting the use of synoptic report datasets. The level of implementation is influenced significantly by whether the application is centralized or locally implemented in an LIS. Ellis and Srigley^20^ describe the pathology report’s six reporting levels, ranging from basic (level 1) to advanced and standardized structured reports (level 6). Applications (standalone) or software (integrated into LISs) used for decision making in diagnosis or therapy purposes fall under class IIa according to the European Medical Device Regulations.^21^ At this national level, requirements on interoperability and intraoperability demand the use of advanced standards such as Fast Healthcare Interoperability Resources (FHIR) and open Electronic Health Record (openEHR).^22^^,^^23^

Unfortunately, both at the individual level and at the national level, synoptic reporting is still not adopted by all pathologists. Pathology and radiology reports parallel each other to some extent.^24^ In radiology, structured reporting is increasingly being used, specifically in the setting of multiparametric magnetic resonance imaging reports, and clinical disciplines are following,^25^ but the global level of implementation, as in pathology, is also far from being universal.^26^

Pathology has been identified as a strong candidate for AI development, particularly in the field of cancer diagnosis (potentiates early-stage diagnosis, refines disease classification, shortens turnaround time) and tissue biomarker analytics (quantification for precision and prediction of outcomes).^27^^,^^28^ During the clinical exercise of Pathology occurs the generation of data necessary for training AI algorithms, either images or report contents, that are required for the deployment of AI tools. Synoptic reports play a crucial role by creating databases with standardized templates, facilitating the acquisition of structured data of a consistent quality ready to train AI algorithms. This structured approach ensures that algorithms receive the cleanest data possible, thereby accelerating the aforementioned processes at significantly higher rates. In essence, synoptic reports may act as a catalyst, streamlining the flow of information to AI systems and optimizing their performance.

As we navigate the intersection between AI and pathology, it becomes evident that the meaningful message conveyed through synoptic reports is no longer confined to the understanding of pathologists and oncologists or the direct benefit to patients. The convergence of technology, structured data, and AI propels the field of pathology toward a future where the synthesis of information benefits not only health care professionals and patients but also serves as a wellspring of knowledge for machines, paving the way for unprecedented strides in data mining and health care innovation, and so creating a quality loop.
